# Improving Hypoxia Adaption Causes Distinct Effects on Growth and Bioactive Compounds Synthesis in an Entomopathogenic Fungus *Cordyceps militaris*

**DOI:** 10.3389/fmicb.2021.698436

**Published:** 2021-06-22

**Authors:** Ying Wang, Zhanshan Yang, Dapeng Bao, Bo Li, Xin Yin, Yingying Wu, Hongyu Chen, Guirong Tang, Nanyi Li, Gen Zou

**Affiliations:** ^1^National Engineering Research Centre of Edible Fungi, Key Laboratory of Edible Fungi Resources and Utilization (South), Ministry of Agriculture, Institute of Edible Fungi, Shanghai Academy of Agricultural Sciences, Shanghai, China; ^2^Department of Horticulture, College of Agriculture and Food Science, Zhejiang A&F University, Lin’an, China; ^3^CAS Key Laboratory of Insect Developmental and Evolutionary Biology, CAS Center for Excellence in Molecular Plant Sciences, Shanghai Institute of Plant Physiology and Ecology, Chinese Academy of Sciences, Shanghai, China

**Keywords:** *Cordyceps militaris*, *Vitreoscilla* hemoglobin, SREBP, hypoxia, bioactive compounds

## Abstract

*Cordyceps militaris* is an entomopathogenic fungus producing a variety of bioactive compounds. To meet the huge demand for medicinal and edible products, industrialized fermentation of mycelia and cultivation of stromata have been widely developed in China. The content of bioactive metabolites of *C. militaris*, such as cordycepin, is higher when cultivated on silkworm pupae than on rice or in broth. However, compared with other cultivation methods, *C. militaris* grows more slowly and accumulates less biomass. The hypoxic environment in pupa hemocoel is one of environmental factor which is not existed in other cultivation methods. It is suggested that hypoxia plays an important role on the growth and the synthesis of bioactive compounds in *C. militaris*. Here, we demonstrated that the distinct effects on the growth and synthesis of bioactive compounds employing different strategies of improving hypoxia adaption. The introduction of *Vitreoscilla* hemoglobin enhanced growth, biomass accumulation, and crude polysaccharides content of *C. militaris*. However, cordycepin production was decreased to 9–15% of the control group. Meanwhile, the yield of adenosine was increased significantly. Nonetheless, when the predicted bHLH transcription factor of sterol regulatory element binding proteins (SREBPs) was overexpressed in *C. militaris* to improve the hypoxia adaption of fungal cells, cordycepin content was significantly increased more than two-fold. These findings reveal the role of SREBPs on growth and bioactive compounds synthesis. And it also provides a scientific basis for rationally engineering strains and optimization strategies of air supply in cultivation and fermentation.

## Introduction

*Cordyceps militaris* is a well-known edible and medicinal mushroom, and has an extensive popularity as a traditional Chinese medicine for a long time in China (Paterson, 2008; Zhou et al., 2009; Reis et al., 2013). Compared to *Ophiocordyceps sinensis*, another medicinal fungus belonging to the genus *Cordyceps*, *C. militaris* is much easier to cultivate (Wu et al., 2020). Industrialized cultivation and fermentation of *C. militaris* have been successfully realized (Lou H. et al., 2019). Therefore, the market price of *C. militaris* is far lower than that of *O. sinensis* (Yin et al., 2018). Currently, it has been developed into a variety of commercial products, since its biomass is rich in bioactive compounds. Therefore, *C. militaris* has more promising application prospects (Wang et al., 2017).

Previous studies have proved that *C. militaris* contains a variety of bioactive compounds beneficial to human body, such as cordycepin, *Cordyceps* polysaccharides, oxalic acid, carotenoid, and pentostatin (Xia et al., 2017; Nurmamat et al., 2018; Kunhorm et al., 2019; Lou H.W. et al., 2019; Chen et al., 2020). Among them, cordycepin is the most studied and has been confirmed to interfere with RNA synthesis, inhibit cell proliferation, and have anti-cancer effects (Cunningham et al., 1950; Lee et al., 2013). At present, the fruiting bodies of *C. militaris* have been commercialized for medicinal and healthcare products. Even as an ordinary edible mushroom, it is used for a food ingredient of Chinese cuisine. In addition to fruit bodies grown on grains or silkworm pupae, mycelia in submerged culture have become important raw materials for industrial application of *C. militaris* (Chan et al., 2015). At all events, the content of cordycepin is the main evaluation index of its quality (National industry indicators of China, GHT 1240-2019) (Li et al., 2019). Different cultivation methods lead to different levels of cordycepin (Dong et al., 2014; Guo et al., 2016). In addition to the nutrient content of the culture medium, environmental factors substantially affect the synthesis of cordycepin (Wang et al., 2017; Lou H. et al., 2019; Suparmin et al., 2019). It is essential to clarify the regulatory mechanisms of these environmental factors for large-scale industrial production of high-quality *C. militaris* fruiting bodies and mycelia (Dong et al., 2015).

Compared to stirred fermentation, static fermentation contributes to a higher yield of cordycepin (Suparmin et al., 2019). When *C. militaris* is cultured in a liquid medium (static culture), the hypha on the surface grows vigorously and forms a layer of aerial mycelia. As a result, the submerged hyphae were isolated from the air and grew toward the bottom of the culture medium. A hypoxic environment appears in the submerged mycelia because of the liquid surface culture (van Keulen et al., 2003). It was confirmed that submerged mycelia contribute to the production and secretion of cordycepin in the media instead of the hypha on the surface (Suparmin et al., 2017, 2019). It suggests that a hypoxic environment may induce the synthesis and secretion of cordycepin. In addition, it is possible that the hypoxic environment of insect hemocoel also caused the higher cordycepin content in the fruiting bodies growing on pupae (Kato et al., 2021). However, the regulatory mechanism of hypoxia in bioactive compounds synthesis has not been investigated in *C. militaris*. Alleviating hypoxic stress in *C. militaris* cells is an effective way to comfirm our hypothesis. The prokaryotic hemoglobin (*Vitreoscilla* hemoglobin; VHb) from the obligate aerobic bacterium *Vitreoscilla* is an oxygen-binding protein, acting as an O_2_ conveyor and transporter (Wakabayashi et al., 1986). The transformation of VHb has been confirmed to efficiently relief hypoxia stress in bacteria (Horng et al., 2010), yeasts (Wu and Fu, 2012), plants (Jokipii et al., 2008), and animals (Pendse and Bailey, 1994). Recently, many reports have verified that this strategy also works as an O_2_ conveyor and transporter in filamentous fungi (Lin et al., 2017; Wang et al., 2019; Xu et al., 2019). Sterol regulatory element binding proteins (SREBPs) are also required for adaptation to hypoxic environment in fungi (Bien and Espenshade, 2010; Gutierrez et al., 2019). Currently, SREBP-like orthologs have been identified in a great many of fungi, especially *Pezizomycotina*, which comprises many pathogenic fungal species of animal and plant pathogens (Ruan et al., 2019). In addition to their role in hypoxia adaptation, these proteins are essential for the pathogenesis and tolerance to antifungal agents (Hillmann et al., 2015; Burgain et al., 2019). However, the functions of hypoxia and its regulators have not been well investigated in *C. militaris*. In a previous study, only increased expression levels of the genes involved in the ergosterol biosynthetic pathway were reported in hypoxic submerged mycelia, but not of genes encoding SREBPs (Suparmin et al., 2019). Therefore, the investigation of exogenous *vgb* and endogenous SREBPs-related genes is helpful to better understand the regulatory role of hypoxia stress in the growth and biosynthesis of bioactive compound in *C. militaris*.

In this study, we employed the VHb to relieve hypoxia in *C. militaris*, and analyzed the growth and the main metabolites of VHb-transformed strains. We also constructed an SREBP-overexpressing strain for comparing the effects of different strategies to improve hypoxia adaptation on *C. militaris*. We thus engineered a high-yield polysaccharide and fast-growing strain, and also constructed a strain with high cordycepin fermentation level. Our research also provides a good optimization strategy for large-scale artificial cultivation and fermentation.

## Materials and Methods

### Strain, Media, and Culture Conditions

The wild-type (WT) strain of *C. militaris* (CM01) was a gift from Prof. Wang (CAS Center for Excellence in Molecular Plant Sciences, CAS, China) and was preserved in this laboratory. The fungal strain was cultured in potato dextrose agar (PDA) medium at 25°C for subculture. Mycelia were grown in Sabouraud dextrose broth (SDB) liquid media at 25°C and 150 rpm for 5 days for collection of blastospores and mycelia. *Escherichia coli* strain DH5α (Weidi, Shanghai, China) was cultured in LB medium (yeast extract 5 g/L, tryptone 10 g/L, and sodium chloride 10 g/L) at 37°C for plasmid DNA replication. *Agrobacterium tumefaciens* strain (AGL1), purchased from Sangon Biotech Co., Ltd. (Shanghai, China), was used for fungal transformation; it was propagated in LB medium at 28°C.

### DNA Manipulation and Vector Construction

Oligonucleotide primers were synthesized and sequenced by BioSune (Shanghai, China). All molecular cloning procedures, including genomic DNA extraction, DNA fragment acquisition, restriction-ligase reaction, transformation, colony verification, plasmid propagation, and sequencing, were operated according to the previous report (Wang et al., 2020; Zou et al., 2020). The Pgpd promoter, Ptef promoter, and Sre1N encoding gene were amplified from the genomic DNA of CM01. The VHb encoding gene (*vgb*) of *Vitreoscilla* was synthesized using GenScript (Nanjing, China) (Supplementary Data 1). The primer pairs used are shown in Supplementary Table 1. The plasmids of Pxbthg-Pgpd-VHb, Pxbthg-Ptef-VHb, and Pxbthg-oeSre1N were obtained by linking the target fragment with the expression vector of Pxbthg, digested by *Hin*dIII and *Bam*HI using a one-step rapid cloning kit (Yeasen, Shanghai, China).

### Transformation and Screening

*Agrobacterium-*mediated transformation was based on previously described methods (Wang et al., 2020; Zou et al., 2021). The mycelia of CM01 were cultured in SDB at 25°C and 150 rpm for 4 days. Blastospores were collected using sterile non-woven fabric and diluted to 10^7^–10^8^ spores/mL. Transformants were screened using M-100 medium (KH_2_PO_4_ 16 g/L, Na_2_SO_4_ 4 g/L, KCl 8 g/L, MgSO_4_⋅7H_2_O 2 g/L, CaCl_2_ 1 g/L, and M-100 trace element solution 8 mL/L. M-100 trace element solution: H_3_BO_3_ 0.06 g/L, MnCl_2_⋅4H_2_O 0.14 g/L, ZnCl_2_ 0.40 g/L, Na_2_MoO_4_⋅2H_2_O 0.04 g/L, FeCl_3_⋅6H_2_O 0.10 g/L, CuSO_4_⋅5H_2_O 0.40 g/L) containing 50 μg/mL cefotaxime sodium and 150 μg/mL hygromycin B. Genomic DNAs were extracted for transformant verification. The primers used are listed in Supplementary Table 1. Western blots were used to analyze the expression of the VHb protein. Three transformants were randomly selected for following tests. Mycelia were collected and ground using 1 mL lysis buffer for WB/IP assays (Yeasen, Shanghai, China) and 5 μL PMSF protease inhibitor (Yeasen). The sample was decomposed on ice for half an hour and then centrifuged (12,000 × *g*, 4°C) for 5 min. The VHb was verified using a 6 × His-tag and HA-tag monoclonal antibody (Yeasen).

### Growth Assay

One milliliter of blastospore suspension (∼10^5^ spores) was inoculated onto a 250-mL flask and incubated at 25°C, 150 rpm. The mycelia were filtered, washed, dried, and weighed. To measure the growth on plates, 1 μL of blastospore suspension (∼10^4^ spores) inoculated onto PDA plate and incubated at 25°C. The growth diameter was measured. Chinese Tussah silkmoth (*Antheraea pernyi*) pupae were used for fruiting body cultivation. Briefly, blastospore suspension (10 μL) of WT strain and its transformants (5 × 10^6^ blastospores/mL) was injected into 5-day-old *A. pernyi* pupae. The injected pupae were incubated at 25°C (12:12-h dark/light, >95% relative humidity) for fruiting bodies formation. The growth status of *C. militaris* on pupae was observed regularly in 50 days.

### Analysis of Adenosine, Cordycepin, and Polysaccharides

To measure the yield of adenosine and cordycepin, the WT strain and its transformants were incubated in 50 mL SDB for 4 days at 25°C, 150 rpm. The cultures were then incubated statically for 11 days. The cultured mixtures were divided into supernatants and mycelia by filtration. The supernatants were further filtered using membrane filter (0.25 μL, Pall; Ann Arbor, MI, United States) for the detection of bioactive compounds. The collected mycelia were freeze-dried after washed three times with distilled water. And then the dried mycelial granules were ground into powder in liquid N_2_. The powdery mycelia were extracted using deionized water (1:20, w:v) and sonicated at 40 KHz and 225 W for 1 h. The yield of adenosine and cordycepin were determined via high-performance liquid chromatography analysis using Waters Alliance e2695 (Waters, MA, United States) with a Waters SunFire^®^ C-18 reverse phase column (100 Å, 5 μm, 4.6 mm × 250 mm; MA, United States). The standard adenosine (Catalog No. A9251, Sigma) and cordycepin (Catalog No. C3394, Sigma) were used for standard curves. The elution conditions were modified for adenosine and cordycepin with a solvent of methanol and deionized water (1:4, v:v). The retention time of aimed products was monitored was at a wavelength of 260 nm (flow rate: 1 mL/min; column oven: 25°C).

Crude polysaccharides of the transformants and WT strains were extracted using an improved water extraction method (Cui et al., 2019). Briefly, the mycelial biomass was separated from the fermentation mixturesby filtration. The filtered broth was centrifuged at 10,000 × *g* for 10 min and was collected for determining the extracellular polysaccharides. And the collected mycelia were freeze-dried for determining mycelial polysaccharides, after washing three times with distilled water. Extracellular polysaccharides were deposited by mixing the fermentation broth absolute ethanol (1:4, v:v). The precipitate was obtained after centrifuge at 12,000 × *g* for 20 min, and then was freeze-dried. Mycelial polysaccharides were extracted from the collected mycelial biomass at 100°C for 2 h. The extract was mixed with 4 volumes of absolute ethanol and was deposited for 24 h at 4°C, and finally was freeze-dried and weighted. The production of crude polysaccharide was calculated by dividing the weight of polysaccharide by the volume of fermentation broth or by the freeze-dried mycelial biomass.

### Quantitative Real Time PCR Analysis of Transformants

Total RNA was extracted using a Redzol kit (SaiBaiSheng, Shanghai, China). The total RNA mass and concentration were verified using 1% agarose gel electrophoresis and a Nanodrop1000, respectively. PrimeScript^TM^ RT reagent kit (with genomic DNA eraser) (Takara, Dalian, China) was used for reverse transcription of cDNA, according to the manufacturer’s instructions. Quantitative real-time PCR (qRT-PCR) was carried out using TB Green^®^ Premix Ex Taq^TM^ II (Tli RNaseH Plus) (Takara) and SYBR^®^ Green Reagents (Takara). The relative expression level of genes involved in cordycepin biosynthesis (*cns1*: CCM_04436, *cns2*: CCM_04437) and predicted sterol regulatory element-binding proteins (*sre1n*: CCM_04014, *scp1*: CCM_03924, *ins1*: CCM_07354, *ofd1*: CCM_07850) were quantified using qRT-PCR. The *sre1* orthologs were discarded in the genome assembly process and were re-corrected at Scaffold 00003: 3173021-3176010.^1^ The oligonucleotide primers used are listed in Supplementary Table 1. The β-tubulin gene (CCM_07292) was used as an endogenous control to quantify the relative gene expression. Relative gene expression levels were calculated using the 2^–Δ^ ^Δ^ *^*CT*^* method.

### Data Processing

The two-tailed Student’s *t*-test was analyzed using Prism 5.0 (Graphpad, San Diego, CA, United States) and Microsoft Excel 2016 (Redmond, WA, United States) for statistical analysis (^∗^*P* < 0.05; ^∗∗^*P* < 0.01; ^∗∗∗^*P* < 0.001). All experiments and tests were performed in triplicates.

## Results

### Heterologous Expression of the VHb Gene in Transformants

**FIGURE 1:**
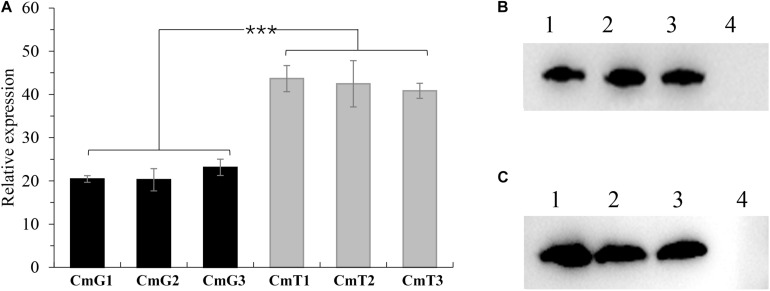
Verifications of *vgb*-expression in *C. militaris* transformants. **(A)** Comparison of relative expression levels of *vgb* in transformants. CmG1-3: *vgb* expressed tranformants controlled by Pgpd promoter; CmT1-3: *vgb* expressed tranformants controlled by Ptef promoter. Significant differences between transformants of two series (Student’s *t*-test): ***P < 0.001 **(B)** Western blots of *vgb* expression using Pgpd promoter. Lane 1–3: transformants CmG1-3; lane 4: wild type strain CM01. **(C)** Western blots of *vgb* expression using Ptef promoter. Lane 1–3: transformants CmT1-3; lane 4: wild type strain CM01. Error bars show standard deviation of three replicates.

To study the regulation of hypoxia, we first attempted to investigate phenotypic changes after introducing *vgb* (Supplementary Table 1) into *C. militaris* to alleviate hypoxia. VHb, a homodimeric oxygen-binding protein encoded by *vgb*, can enhance the oxygen utilization of a cell (Frey and Kallio, 2003), thereby improving cellular respiration efficiency. We utilized two native promoters (Ptef, promoter of translation elongation factor 1α coding gene (CCM_00809), and Pgpd, promoter of glyceraldehyde-3-phosphate dehydrogenase encoding gene (CCM_04549) (Zheng et al., 2011) with different strengths to control VHb expression. The constructed plasmids Pxbthg-Ptef-VHb (under control of Ptef) and Pxbthg-Pgpd-VHb (under control of Pgpd) were transformed into *C. militari*s CM01 strain (Wang et al., 2020). The randomly selected transformants were designated as CmT1–CmT3 and CmG1–CmG3. All the selected transformants were showed that *vgb* was successfully transformed (Supplementary Figure 1). After 10 days of incubation in SDB, total RNA and intracellular protein were extracted for further verification of *vgb* transcription and translation (Wang et al., 2020). Using RT-qPCR the relative expression levels of *vgb* were consistent with the strength of the corresponding promoter (Figure 1A; Zheng et al., 2011). The copy numbers of *vgb* in the genome of the triplicate transformants were identical based on the expression levels (Figure 1A). Relative expression levels of *vgb* in transformants CmT1–CmT3 were about two-fold higher than those in CmG1–CmG3 (Figure 1A). The western blot assay based on intracellular protein confirmed that VHb protein was correctly expressed in each transformant (Figures 1B,C and Supplementary Figure 2). These results indicate that the VHb protein can be correctly expressed in all randomly selected transformants.

### VHb Expression Improves Mycelial Growth

**FIGURE 2:**
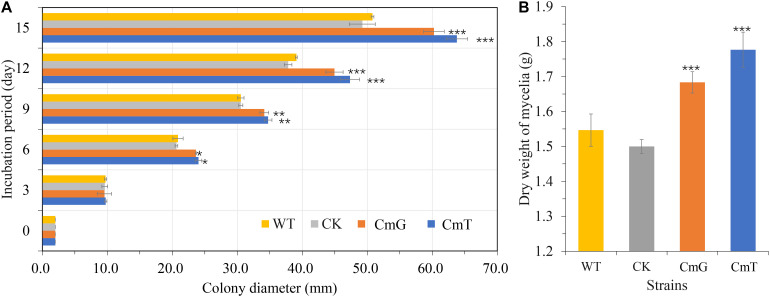
Effects of VHb on growth of *C. militaris*. **(A)** Colony diameters of *C. militaris* and its tranformants growing on PDA plates. WT: CM01; CK: transformation control; CmG: average diameter of transformants Cmpgd1-3; CmT: average diameter of transformants CmT1-3. **(B)** Dry weight of mycelial biomass of *C. militaris* and its tranformants growing in SDB for 15 days. WT: CM01; CK: transformation control; CmG: average dry weight of mycelia biomass of transformants CmG1-3; CmT: average dry weight of mycelia biomass of transformants CmT1-3. Error bars show standard deviation of three replicates. Significant differences between CM01 and transformants (Student’s *t*-test): *P < 0.05, **P < 0.01, ***P < 0.001.

In filamentous fungi, heterologous expression of *vgb* promotes growth and increases biomass accumulation (Roos et al., 2002). In the present study, colony diameter (Figure 2A) was measured in three replicates of transformants expressing *vgb*, the transgenic strain (CK, the strain transformed using the Pxbthg plasmid without *vgb* cassette), and the WT to observe the effect of VHb on PDA plates. Our results showed that *vgb* expression enhanced growth significantly after 6 days of incubation at 25°C. The fastest growing transformants (CmT1–CmT3), which were under the control of Ptef, increased by about 20% in colony diameter compared to the WT strain on the 15th day (Figure 2B). We also tested the amount of mycelia under liquid culture conditions. After 15 days of incubation, the dry weight of mycelia of the transformants increased by 12.2% (CmG1–CmG3, *P* < 0.001) and 18.4% (CmT1–CmT3, *P* < 0.001), compared to that of the CK and WT strain. These results demonstrate that VHb expression promotes mycelial growth in *C. militaris*.

### VHb Expression Promotes the Fruiting Body Formation

**FIGURE 3:**
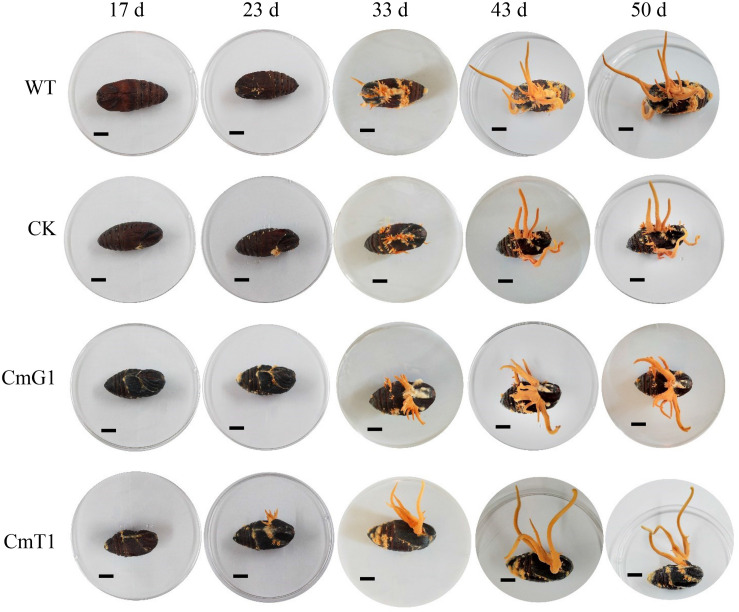
Effects of VHb on fruiting body formation. Fruiting body formation was observed on day 17, 23, 33, 43, and 50, after injection using blastospores. WT: CM01; CK: transformation control; CmG1: transformant Cmpgd1; CmT1: transformant CmT1.

Although the mycelia of *C. militaris* are often developed into various health products, its fruiting body is more commonly utilized as an edible mushroom. To determine the influence of VHb on fruiting body formation, *A. pernyi* pupae were used as cultivation substrate to grow *C. militaris* strains. Suspension spore solution was added to each pupa and cultured in an incubator at 25°C (L:D = 12:12). After 17 days, stromata formed at the surface of the pupae injected with blastopores of CmG1 and CmT1 strains; however, no stroma was observed on the spores of WT and CK strains (Figure 3). When cultured for 23 days, the formation of fruiting bodies was observed in all pupae, and those injected with CmT1 spores had grown to approximately 1 cm. However, WT formed fruiting bodies of approximately 1 cm in length at 33 days. At the moment, the fruiting bodies expressing the VHb protein had grown to 2 cm (CmG1) to 4 cm (CmT1). Up to 50 days, the fruiting bodies of the *vgb-*expressing transformants had obvious advantages in terms of length (Figure 3) and weight (Supplementary Table 2). These results showed that the introduction of VHb allowed fruiting bodies to form rapidly and grow vigorously. Compared with CmG1, the CmT1 strain has more obvious advantages. This is consistent with the relative expression levels of *vgb*. The relative expression levels of *vgb* in CmT1 were about 1.1 times higher than those of CmG1 (Figure 3). This is similar to the result of mycelial growth in the PDA plate or the SDB shake flask (Figure 2). These results indicate that alleviating hypoxia can effectively promote growth and biomass accumulation. VHb has been reported to promote the production of chitinase and other proteases (Zhang et al., 2014). This may also lead to the earlier formation of fruiting bodies in transformants.

### Hypoxia Influences Yield of the Main Bioactive Metabolites in *C. militaris*

**FIGURE 4:**
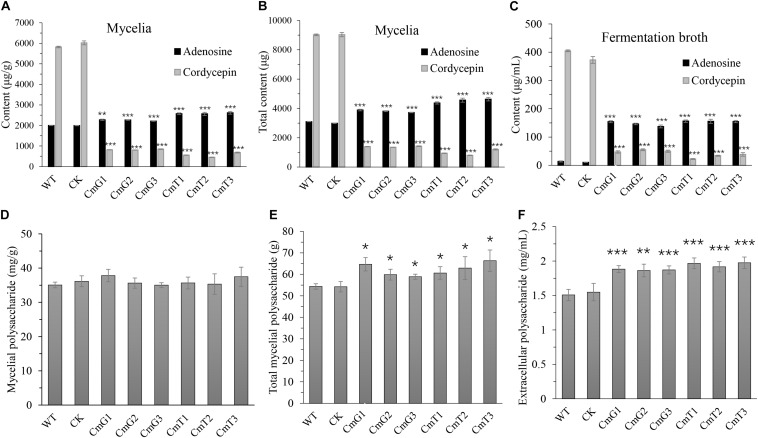
Comparisons of yield of the main bioactive compounds of *C. militaris* and its *vgb*-expressed transformants after 15 days’ fermentation. **(A)** Content of mycelial adenosine and cordycepin. **(B)** Total content of mycelial adenosine and cordycepin. **(C)** Content of extracellular adenosine and cordycepin in fermentation broth. **(D)** Crude mycelial polysaccharides. **(E)** Total crude mycelial polysaccharides in 50-mL fermentation broth. **(F)** Crude exo-polysaccharide in fermentation broth. WT: CM01; CK: transformation control; CmG1-3 and CmT1-3: *vgb*-expressed transformants. Error bars show standard deviation of three replicates. Significant differences between CM01 and transformants (Student’s *t*-test): *P < 0.05, **P < 0.01, ***P < 0.001.

*Vitreoscilla* hemoglobin expression stimulates the yield of secondary metabolites (Li et al., 2016; Xu et al., 2019). We also tested the main bioactive metabolites of *C. militaris*, including cordycepin, adenosine, and polysaccharides. Unlike the reports in other microorganisms, cordycepin, the main metabolite in *C. militaris*, was significantly reduced in the transformants. Both intracellular (mycelia) and extracellular (fermentation broth) cordycepin of transformants decreased to 9–15% of that of the control group (Figure 4). The cordycepin concentration (both extracellular and intracellular) of CmT1–CmT3 was lower (47–85%) than that of CmG1–CmG3 (Figure 4). In contrast, the adenosine levels of the transformants increased overall. The intracellular adenosine concentration and the total intracellular denosine increased by approximately 10–25% and 20–49%, respectively (Figures 4A,B); and the extracellular adenosine concentration increased by more than 10 times (Figure 4C). Moreover, the content of adenosine and cordycepin in the fruiting bodies harvested on the 50th day also showed that there was a large amount of adenosine accumulated in transformants, and the cordycepin content decreased to undetectable level (Supplementary Figure 3).

The mycelial polysaccharide content did not differ among the tested strains (Figure 4D). However, since the transformants accumulated more mycelial biomass (Figure 4E), the total amount of mycelial polysaccharides was about 10–20% higher than that of the WT strain and transformation control. The yield of extracellular polysaccharides increased significantly by 24–30% in the fermentation broth of transformants, compared to that in the control group (Figure 4F). These results suggest that the synthesis of polysaccharides and cordycepin has different oxygen requirements. In this study, the increased content of crude polysaccharides may be related to the enhanced mycelial biomass after alleviating hypoxia. The synthesis of secondary metabolites, such as cordycepin, may be related to the adaptation of fungi to environmental stress. However, under the same conditions, the critical decline of cordycepin yield suggests that hypoxia might be essential for stimulating cordycepin synthesis.

### SREBPs Involved in Cordycepin Gene Cluster Expression

To investigate the decrease in cordycepin production, we detected the relative expression of genes in the cordycepin biosynthesis cluster. The results showed that the expression levels of *cns1–cns2* in the transformants were significantly reduced to less than 1.4% (CmT1–CmT3) or 5.7% (CmG1–CmG3) of the control strain (Supplementary Figure 4). The expression level of *vgb* was negatively correlated with that of the cordycepin gene cluster (Figure 1A). This suggested that the improvement in the oxygen utilization rate determined the decrease in cordycepin production. In contrast, the activation of cordycepin gene cluster expression might be related to hypoxia stress in *C. militaris* cells. It was reported that SREBPs required for hypoxia fitness in fungi (Dhingra and Cramer, 2017). Through bioinformatics analysis, we found SREBP orthologs in *C. militaris*, including Sre1N (CCM_04014), Scp1 (CCM_03924), Ins1 (CCM_07354), and Ofd1 (CCM_07850). However, Sre1 ortholog was discarded in the genome assembly process because of the internal gap in sequence (see text footnote 1). Consequently, the expression levels of SREBPs in the genome were determined. It was found that all the SREBP-encoding genes were downregulated by 40–70% in the transformants (Supplementary Figure 5). These results suggest that the introduction of VHb alleviates hypoxia stress in the cells.

Regulation of SREBPs in fungi is complex and involves additional regulatory layers including post-translational mechanisms, however it was critically controlled by levels of Sre1 and its orthologs (Dhingra and Cramer, 2017). To confirm our hypothesis, we overexpressed the gene encoding Sre1N, i.e., one of bHLH transcription factors in SREBPs, considering the incorrect annotation of Sre1. Randomly selected transformants designated as OeSre1N1-3. In *sre1n*-overexpressing transformants, the expression levels of *sre1n* significantly inscreased by 11–16-fold. This indicated that the *sre1n* gene was successfully overexpressed in the three randomly selected transformants (Supplementary Figure 6). Meanwhile, *ofd1* was also up-regulated by more than two-fold. Ofd1 was reported negatively regulated Sre1N levels in a proteasome dependent manner (Hughes and Espenshade, 2008). This may be due to feedback regulation triggered by too much Sre1N in the cells. All the other SREBP-encoding genes were significantly downregulated. It was similar to the introduction of *vgb* gene (Supplementary Figure 6). Under hypoxic stress, cells overcome the harsh environment by upregulating the expression of these genes (Hughes and Espenshade, 2008). Therefore, down-regulation of these genes indicates that transformants have better adaptability to hypoxic environment, after overexpression of *sre1n*.

**FIGURE 5:**
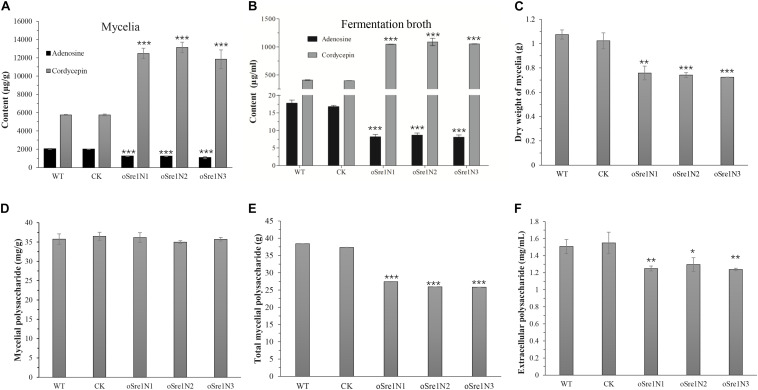
Comparisons of yield of the main bioactive compounds and growth of *C. militaris* and its *sre1n*-overexpressed transformants after 15 days’ fermentation. **(A)** Content of mycelial adenosine and cordycepin. **(B)** Content of extracellular adenosine and cordycepin in fermentation broth. **(C)** Dry weight of mycelial biomass of *C. militaris* and its tranformants growing in SDB for 15 days. **(D)** Crude mycelial polysaccharides. **(E)** Total crude mycelial polysaccharides in 50-mL fermentation broth. **(F)** Crude exo-polysaccharide in fermentation broth. WT: CM01; CK: transformation control; Sre1N1-3: randomly selected *sre1n*-overexpressed transformants. Error bars show standard deviation of three replicates. Significant differences between CM01 and transformants (Student’s *t*-test): *P < 0.05, **P < 0.01, ***P < 0.001.

Transformants OeSre1N1-3 and control group were also used to measure the yields of cordycepin, adenosine, and polysaccharides (Figure 5). After 15 days of incubation, the yields of both the extracellular and intracellular cordycepin increased 2.3- and 2.7-fold, respectively, compared to those in the control group (Figures 5A,B). The relative expression levels of *cns1* and *cns2* in OeSre1N1-3 were more than 1.7 times higher than those of control group (Supplementary Figure 7). In contrast, the yield of adenosine significantly decreased to 54 and 48%, respectively (Figures 5A,B). These results indicate that overexpression of *sre1n* can indeed increase cordycepin production. However, the dry weight of transformant mycelia was approximately 70% of that of the WT strain (Figure 5C). And the total mycelial polysaccharide content decreased by 31% (Figures 5D,E). The yield of extracellular polysaccharides significantly decreased by 26–29% in the fermentation broth of transformants, compared to that of the control group (Figure 5F). The decrease in extracellular polysaccharide production may be related to a decrease in mycelial biomass accumulation.

## Discussion

In this study, we found that VHb promoted growth and increased biomass accumulation in *C. militaris*. These results are consistent with those of *Aspergillus sojae* (Mora-Lugo et al., 2015) and *Paecilomyces lilacinus* (Zhang et al., 2014). The yield of crude polysaccharides also increased significantly. These characteristics have many advantages for cost reduction in the cultivation of *C. militaris*. However, the decline in cordycepin is a fatal defect in its quality. This is different from previous reports that VHb can also promote the synthesis of bioactive compounds in other fungi (Arnaldos et al., 2012; Ma and Lin, 2014; Zhang et al., 2014). It is possible that the requirement for oxygen in the growth process may vary in different organisms. However, this verifies our original hypothesis that hypoxia plays an important role in cordycepin production.

The expression levels of all these SREBP-encoding genes in the *vgb*-expressing transformants were decreased simultaneously with those of the cordycepin cluster. In addition, overexpression of Sre1N greatly increased cordycepin production. These results further confirmed our hypothesis that hypoxia stress can activate cordycepin expression. It suggests that uncovering the regulatory mechanism of SREBPs in the synthesis of cordycepin will contribute to optimizing the process of industrial-scale artificial cultivation of *C. militaris*. However, the regulatory pathway of SREBPs are still very superficial in this study, which needs to be further uncovered. In the fission yeast *Schizosaccharomyces pombe*, hypoxia is the major activation signal to proteolyze Sre1 to generate an activated N-terminus (Hughes et al., 2005). This proteolytic process releases the N-terminal transcription factor for nuclear localization and activation of gene expression (Espenshade and Hughes, 2007). It was reported that three Sre1 orthologs, all owning the canonical tyrosine residue in the bHLH DNA binding domain, have their own regulon in *Aspergillus fumigatus*. SrbC (one of Sre1 orthologs without transmembrane and C-terminal domain) is expressed at low levels in conditions examined to date including low oxygen and its role in SREBP gene regulation are under investigation (Chung et al., 2014). In *C. militaris*, there are two Sre1 ortholog. Although Sre1N lacks the predicted transmembrane and C-terminal domains of Sre1, it is confirmed that Sre1N regulates cordycepin synthesis in some way. However, the roles of these two orthologs in SREBP pathway still need to be figured out. Our results could explain the different yields of active compounds under different cultivation conditions. Good aeration in rice medium promotes the growth, development, and synthesis of polysaccharides (Guo et al., 2016). This is consistent with the high oxygen utilization promoted by VHb. When cultivated in pupae, mycelia must compete for oxygen with the cells in the hemocoel of insects. The resulting hypoxia stress promoted the synthesis of cordycepin. This explains why *C. militaris* cultivated in pupae had a high content of cordycepin. In addition, it is consistent with the fact that static submerged liquid fermentation can obtain a higher yield of cordycepin than liquid fermentation with ventilation does (Suparmin et al., 2017, 2019). Static liquid fermentation also induced hypoxia stress in the cells of these submerged mycelia.

In addition, the introduction of VHb and overexpression of Sre1N are both strategies to improve adaptability to hypoxic environments. However, the phenotypes between the two types of transformants are completely different. *Vitreoscilla* hemoglobin effectively increased the mycelial capacity for oxygen utilization, thereby boosting cellular respiration intensity (Suen et al., 2014). Therefore, the *vgb*-expressing transformant cells do not reduce the respiratory efficiency due to the hypoxic environment and then improve their adaptability to the hypoxic environment. On the contrary, SREBPs activate expression of genes encoding enzymes involved in oxygen-dependent metabolic pathways, when hypoxic conditions cause decreased intracellular sterol levels (Chung et al., 2019; Venegas et al., 2020). Fungi sense oxygen levels indirectly through the concentration of specific metabolites, including ergosterol, reactive oxygen species, and unsaturated fatty acids, which are generated only in the presence of O_2_ (Dhingra and Cramer, 2017). Thus, overexpression of Sre1N only increased the efficiency of metabolic pathways but did not substantially improve mitochondrial respiration and redox balance. Nevertheless, these results provide alternative strategies for producing diversity bioactive compounds in cultivation and fermentation.

In conclusion, this study is the first to report the importance of hypoxia stress in the synthesis of cordycepin in *C. militaris*. The successful expression of the functional VHb significantly improved growth and polysaccharide production. In addition, the overexpression of transcription factor in SREBPs enhanced cordycepin yield. Our research revealed the positive and negative regulation of hypoxia during the cultivation of *C. militaris*. These results clearly demonstrate that engineering genes involved in hypoxic stress is an alternative strategy for improving growth and production of bioactive compounds.

## Data Availability Statement

The original contributions presented in the study are included in the article/Supplementary Material, further inquiries can be directed to the corresponding authors.

## Author Contributions

GZ, YW, and DB participated in the conception of the study. YW and ZY carried out the majority of the experiments. XY and BL were involved in measuring growth of *C. militaris*. YYW and HC were involved in different type of HPLC analysis. DB was involved in the project leadership. GT participated in editing the manuscript. GZ and NL were involved in the conception of the study and participated in the guidance with experimental strategies and technical direction. All authors read and approved the final manuscript.

## Conflict of Interest

The authors declare that the research was conducted in the absence of any commercial or financial relationships that could be construed as a potential conflict of interest.

## Footnotes

1 https://mycocosm.jgi.doe.gov/Cormi1/Cormi1.home.html

## Supplementary Material

Supplementary Figure 1 PCR verification of *vgb* gene in transformants.

Supplementary Figure 2 Western blots of *vgb* gene in transformants.

Supplementary Figure 3 Content of adenosine and cordycepin in fruiting bodies.

Supplementary Figure 4 Relative expression levels of genes involved in cordycepin synthesis after introduction of *vgb*.

Supplementary Figure 5 Relative expression levels of genes encoding SREBPs in *vgb*-overexpressing transformants.

Supplementary Figure 6 Relative expression levels of genes encoding SREBPs in *sre1n*-overexpressing transformants.

Supplementary Figure 7 Relative expression levels of genes involved in cordycepin synthesis after overexpression of *sre1n*.

Supplementary Table 1 Oligonucleotides and gene sequences used in this study.

Supplementary Table 2 Biomass of fruiting bodies after 50 days growth.

Supplementary Data 1 Sequence of *vgb* optimized for *C. militaris*.

## Abbreviations

bHLH: basic helix-loop-helix
SREBP: sterol regulatory element binding protein
VHb: *Vitreoscilla* hemoglobin
rt-qPCR: real-time quantitative PCR
WT: wild type
CK: control check
PDA: potato dextrose agar
SDB: Sabouraud dextrose broth.
