# Supplementary material for: Improving Hypoxia Adaption Causes Distinct Effects on Growth and Bioactive Compounds Synthesis in an Entomopathogenic Fungus Cordyceps militaris
Source: Front Microbiol. 2021 Jun 22;12:698436. doi: 10.3389/fmicb.2021.698436 (PMC8258390; doi:10.3389/fmicb.2021.698436)
Supplement: Supplementary Figure 1 — PCR verification of vgb gene in transformants. [file Data_Sheet_1.docx]

Supplementary Material

# Supplementary Data

**Supplementary Data 1**

>*vgb*_optmized_for_*C_militaris*

atgctcgaccagcagaccattaacattatcaaggctaccgtcccagtcctgaaggagcacggcgtcaccatcaccaccaccttctacaagaacctcttcgccaagcaccccgaagtccgcccactcttcgacatgggccgccaggagtccctggaacagcctaaggctctcgctatgaccgtcctggctgctgcccagaacattgagaacctccctgccatcctgccggccgtcaagaagattgccgtcaagcactgccaggctggcgtcgctgctgctcactacccaattgtcggccaggagctcctgggcgccatcaaggaagtcctcggcgacgctgctaccgacgacatcctggacgcttggggcaaggcttacggcgtcattgccgacgtcttcatccaggtcgaggctgacctgtacgctcaggccgtcgaataa

# Supplementary Figures and Tables

## Supplementary Figures


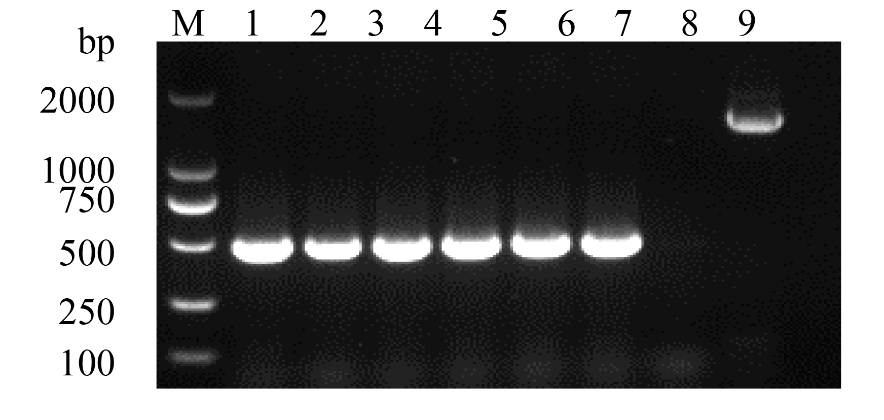


**Supplementary Figure 1.** **PCR verification of *vgb* gene in transformants.** M: marker; 1-3: transformants CmG1-3; 4-6: transformants CmT1-3; 7: wild type CM01, 8: negative control; 9: genomic DNA control of CM01 for amplifying Pgpd promoter.


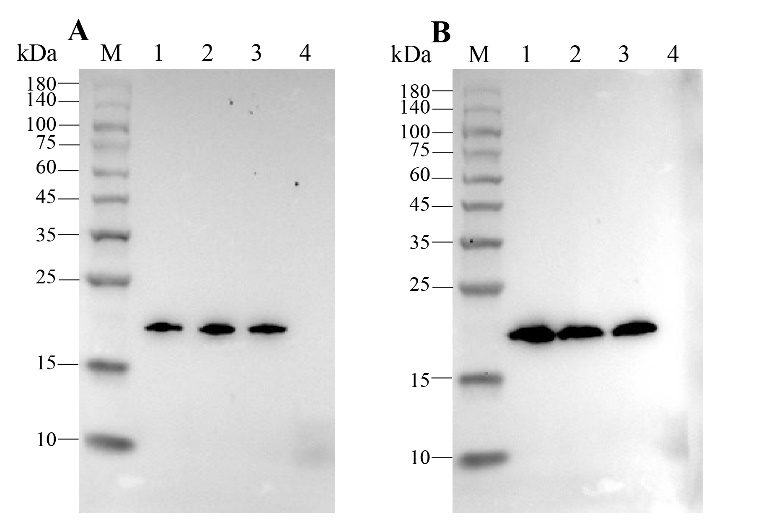


**Supplementary Figure 2.** **Western blots of *vgb* gene in transformants. A:** M: marker; 1-3: transformants CmG1-3; 4: CM01. **B**: M: marker; 1-3: transformants CmT1-3; 4: CM01.


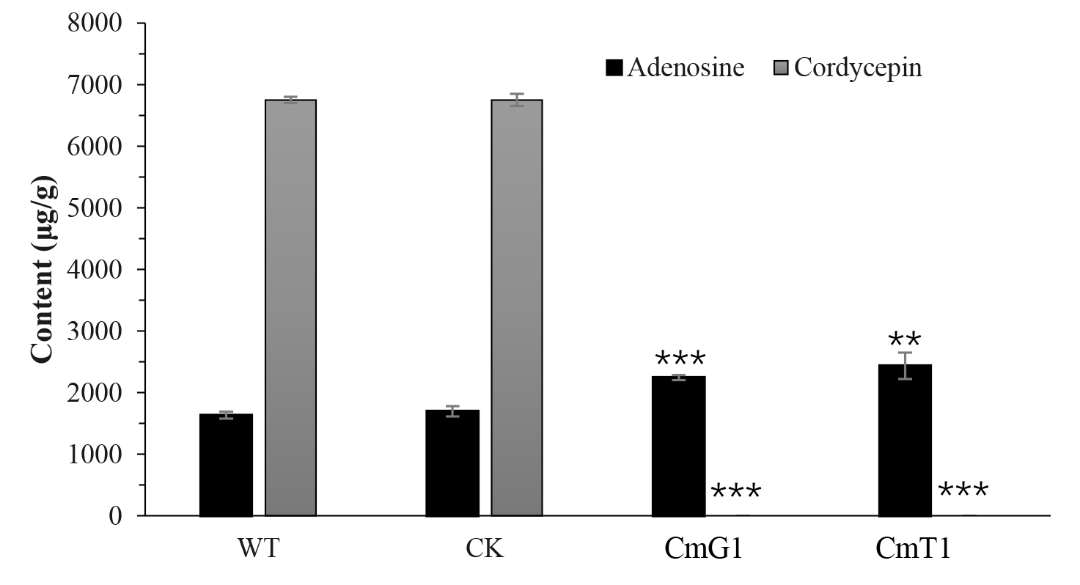


**Supplementary Figure 3.** **Content of adenosine and cordycepin in fruiting bodies**. WT: CM01; CK: transformation control; CmG1 and CmT1: *vgb*-expressed transformants. Error bars show standard deviation of three replicates. Significant differences between CM01 and transformants (Student’s *t*-test): **, *P* < 0.01, ***, *P* < 0.001


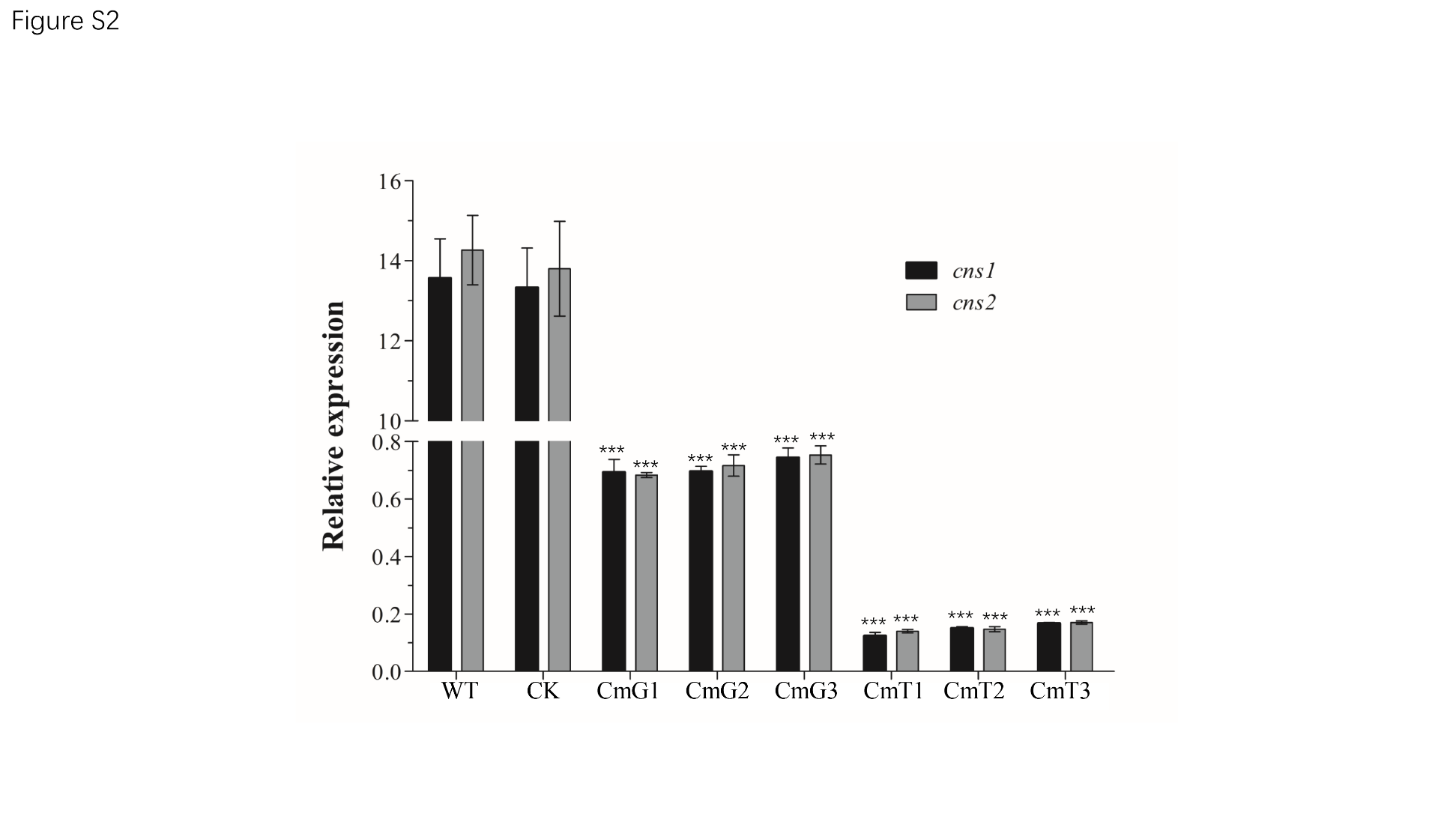


**Supplementary Figure 4.** **Relative expression levels of genes involved in cordycepin synthesis after introduction of *vgb***. *cns1*: gene encoding oxidoreductase domain-containing protein (CCM_04436); *cns2*: gene encoding phosphoribosyl-aminoimidazole-succinocarboxamide synthase (CCM_04437); WT: CM01; CK: transformation control; CmG1-3 and CmG1-3: randomly selected *vgb*-expressed transformants. Error bars show standard deviation of three replicates. Significant differences between CM01 and transformants (Student’s *t*-test): ***, *P* < 0.001


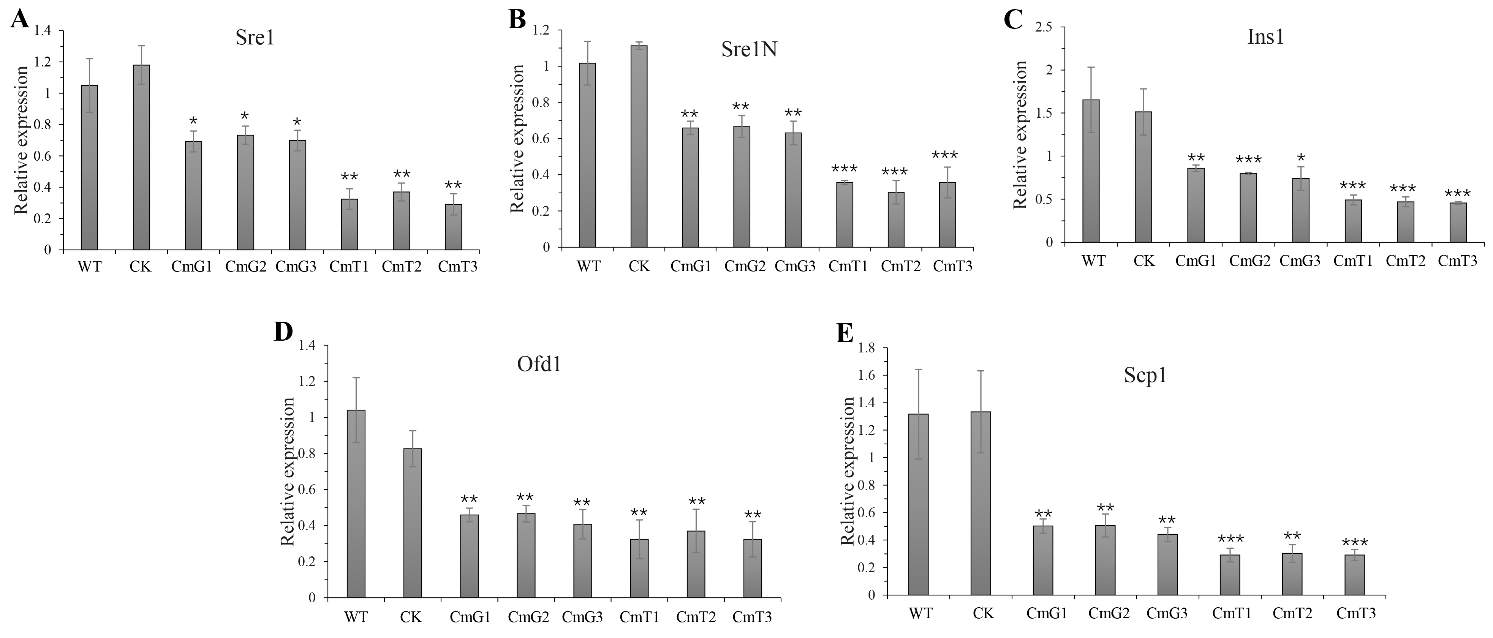
**Supplementary Figure 5.** **Relative expression levels of genes encoding SREBPs in *vgb*-overexpressing transformants**. Sre1: predicted bHLH transcription factor with integral domains (Scaffold 00003: 3173021-3176010,(https://mycocosm.jgi.doe.gov/Cormi1/Cormi1.home.html); Sre1N: the predicted bHLH transcription factor lack of C-terminus (CCM_04014); Scp1: predicted sterol regulatory element-binding protein cleavage-activating protein (CCM_03924); Ins1: predicted INSIG domain protein (CCM_07354); predicted Ofd1: Oxoglutarate/iron-dependent oxygenase degradation domain (CCM_07850); WT: CM01; CK: transformation control; CmG1-3 and CmT1-3: randomly selected *vgb*-expressed transformants. Error bars show standard deviation of three replicates. Significant differences between CM01 and transformants (Student’s *t*-test): *, *P* < 0.05, **, *P* < 0.01, ***, *P* < 0.001


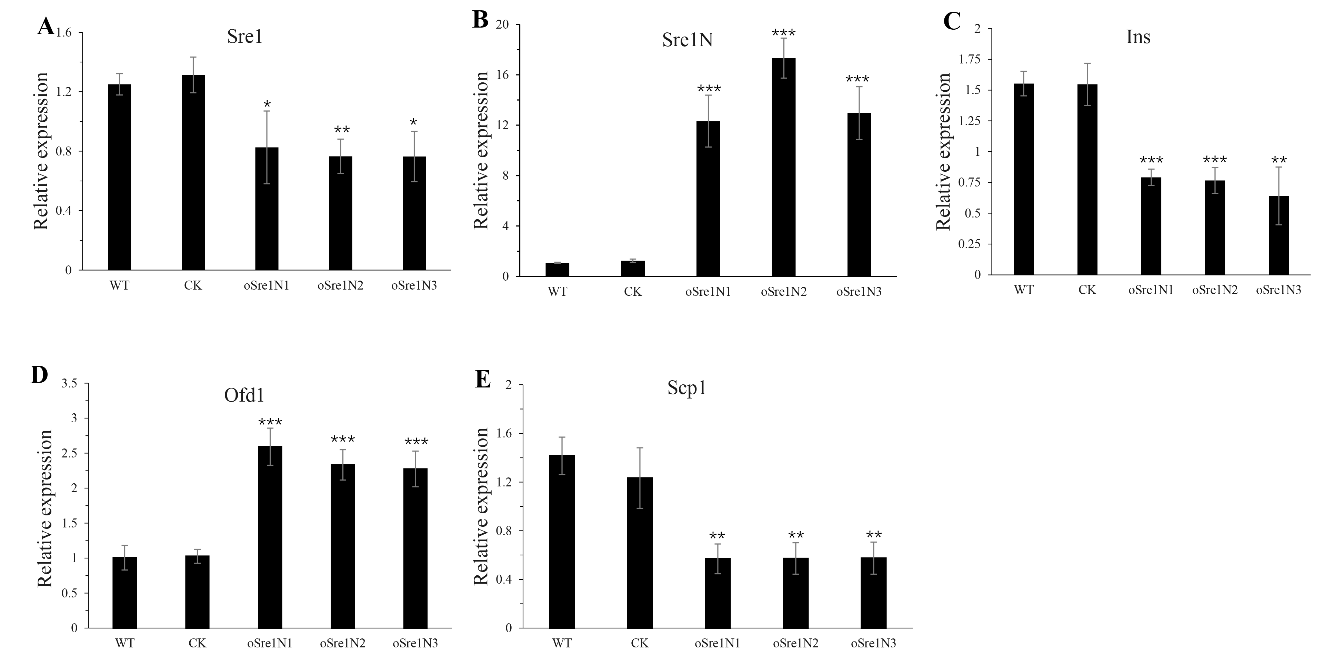


**Supplementary Figure 6.** **Relative expression levels of genes encoding SREBPs in *sre1n*-overexpressing transformants**. Sre1: predicted bHLH transcription factor with integral domains (Scaffold 00003: 3173021-3176010,(https://mycocosm.jgi.doe.gov/Cormi1/Cormi1.home.html); Sre1N: the predicted bHLH transcription factor lack of C-terminus (CCM_04014); Scp1: predicted sterol regulatory element-binding protein cleavage-activating protein (CCM_03924); Ins1: predicted INSIG domain protein (CCM_07354); predicted Ofd1: Oxoglutarate/iron-dependent oxygenase degradation domain (CCM_07850); WT: CM01; CK: transformation control; oSre1N1-3: randomly selected *sre1n*-overexpressing transformants. Error bars show standard deviation of three replicates. Significant differences between Cm01 and transformants (Student’s *t*-test): *, *P* < 0.05, **, *P* < 0.01, ***, *P* < 0.001


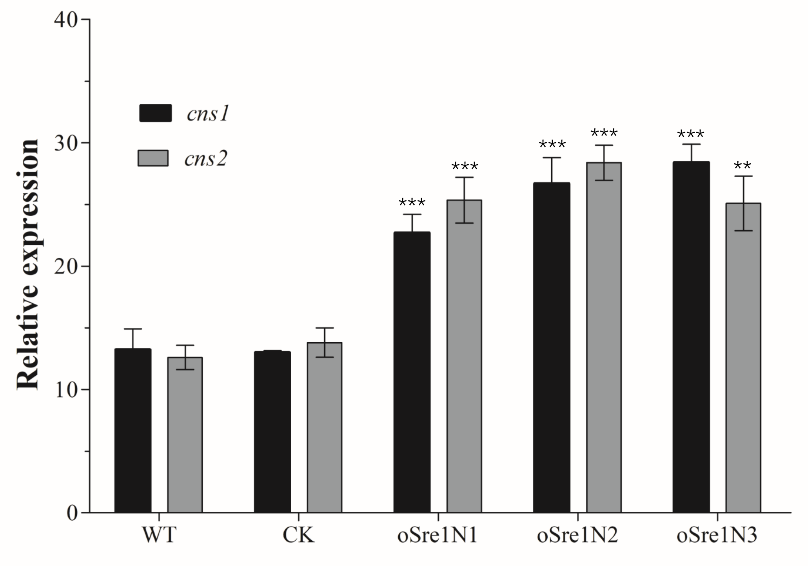


**Supplementary Figure 7.** **Relative expression levels of genes involved in cordycepin synthesis after overexpression of *sre1n***. *cns1*: gene encoding oxidoreductase domain-containing protein (CCM_04436); *cns2*: gene encoding phosphoribosyl-aminoimidazole-succinocarboxamide synthase (CCM_04437); WT: CM01; CK: transformation control; oSre1N1-3: randomly selected *sre1n*-overexpressing transformants. Error bars show standard deviation of three replicates. Significant differences between CM01 and transformants (Student’s *t*-test): **, *P* < 0.01, ***, *P* < 0.001

## Supplementary Tables

**Supplementary Table 1. Oligonucleotides and gene sequences used in this study**

| Names | Sequences (5'-3') | Descriptions |
| --- | --- | --- |
| tefF | tgtaaaacgacggccagtgccaagcttgtcaaggtggccacgacaacc | Primer for amplifying Ptef promoter |
| tefR | aatggtctgctggtcgagcattttgaaggtgtttgttattgaatgcgag |  |
| gpdF | tgtaaaacgacggccagtgccaagcttgaggcaaccgggtcgaatcgga | Primers for amplifying Pgpd promoter |
| gpdR | aatggtctgctggtcgagcattgttcttgattagaaaagtgaggtgagag |  |
| VHbF | atgctcgaccagcagaccatt | Primers for amplifying *vgb* |
| VHbR | ttaatgatgatgatgatgatgttcgacggcctgagcgtaca |  |
| Trp27F | tgtacgctcaggccgtcgaacatcatcatcatcatcattaagcgacacgccatcacg | Primers for amplifying Trp27 terminator |
| Trp27R | gttccctttagtgagggttaattgcgcggatcctgttgaaattaccagcgattcgaaatc |  |
| rtVHbF | gtccgcccactcttcgac | Primers for testing relative expression level of *vgb* |
| rtVHbF | agcctggcagtgcttgac |  |
| rtcns1F | gcgagaagccacttgccttg | Primers for testing relative expression level of *cns1* |
| rtcns1R | ttggttctttggaggccgcc |  |
| rtcns2F | aggtcgctctgacgctgtc | Primers for testing relative expression level of *cns2* |
| rtcns2R | gacctgccacagcaccttg |  |
| rtSre1F | tgcgcatcatgtccaagagt | Primers for testing relative expression level of *sre1* |
| rtSre1R | cctgcatggcgttgttttca |  |
| rtSre1NF | ctgtatccagcaccttcgct | Primers for testing relative expression level of *sre1n* |
| rtSre1NR | gggctggtactctgcttgtt |  |
| rtScpF | gaatcaaggacccaggcaca | Primers for testing relative expression level of *scp1* |
| rtScpR | ggacctcgtctgcctcaaaa |  |
| rtInsF | tcaactcgacgtatctgggc | Primers for testing relative expression level of *ins1* |
| rtInsR | caatggcacgcatcaccaac |  |
| rtOfdF | tccagtacatctcccccacg | Primers for testing relative expression level of *ofd1* |
| rtOfdR | cttccagggcgttgtcttct |  |
| TubF | ttctgagccatcctacgg | Primers for reference gene |
| TubR | cagtcaagggagcaaagc |  |

**Supplementary Table 2.** Biomass of fruiting bodies after 50 d growth

| Strain | WT | CK | CmG1 | CmT1 |
| --- | --- | --- | --- | --- |
| Fresh biomass  (g per chrysalis) | 1.527±0.132 | 1.516±0.236 | 1.829±0.338 | 1.991±0.241 |
| Dry biomass  (g per chrysalis) | 0.215±0.019 | 0.223±0.035 | 0.310±0.117 | 0.362±0.044 |
